# Macrotranscriptomics analysis for decoding the role of *Klebsiella* variicola H8 in aroma compound biosynthesis during fermentation of reconstituted tobacco leaf concentrate

**DOI:** 10.3389/fbioe.2025.1635651

**Published:** 2025-09-09

**Authors:** Yingjie Feng, Wenyuan Qi, Jinchu Yang, Wenzhao Liu, Zongcan Yang, Ke Wang, Duobin Mao, Shen Huang, Tingting Zhang

**Affiliations:** ^1^ Technology Center, China Tobacco Henan Industrial Co., Ltd., Zhengzhou, China; ^2^ School of Tobacco Science and Engineering, Zhengzhou University of Light Industry, Zhengzhou, China

**Keywords:** metatranscriptome, reconstituted tobacco leaf concentrate, glycoside hydrolases, aroma-enhancing compounds, microbial fermentation

## Abstract

Microbial fermentation shapes the reconstituted tobacco leaf concentrate’s (RTLC) chemical composition and sensory quality. This study employed macrotranscriptomic analysis to investigate how the aroma-enhancing bacterium *Klebsiella variicola* H8 modulates RTLC fermentation. High-throughput second-generation RNA sequencing revealed that the transcript abundance of *K. variicola* H8 increased from 5.92% at the start of fermentation to 14.78% at 16 h, accompanied by the enrichment of other key genera such as *Lactobacillus* and *Citrobacter*. Differential gene expression analysis showed that *K. variicola H8* transcription correlated strongly (R^2^ = 0.85) with water-soluble sugar degradation, while nitrogen and potassium correlations were weaker (R^2^ = 0.47 and 0.41, respectively). Notably, the upregulation of glycoside hydrolases-particularly GH78, GH13_25, GH31, and GH28-was associated with the release of key non-volatile aroma-enhancing compounds (NAECs), such as β-damascenone (13.24 μg/g), phenylethanol (7.12 μg/g), solanone (5.89 μg/g), dihydrokiwi lactone (6.03 μg/g), and benzyl alcohol (5.15 μg/g). Furthermore, expression levels of apoptosis-related genes increased at 36 h, coinciding with a decline in sensory quality and aroma compound accumulation. These findings reveal the dynamic microbial and enzymatic processes underpinning NAEC production and provide a mechanistic basis for optimizing microbial fermentation in tobacco processing.

## 1 Introduction

Tobacco (*Nicotiana tabacum*) contains ∼5,000 compounds, many of which have been reported to have various pharmacological activities (Banožić et al., 2020). Recently, the extraction of bioactive compounds from plants has attracted significant interest (Suleria et al., 2016; Zou et al., 2021).

Microbial fermentation offers several advantages, such as a high conversion rate, high specificity, and the production of high-quality aroma compounds, and is therefore applied in a wide range of food industries (Hadj Saadoun et al., 2021; Ma et al., 2024; Zara and Fan, 2023). For example, the fermentation of reconstituted tobacco leaves (RTLCs) with *K. variicola* H8 resulted in a ∼25% reduction in nicotine levels and a 45% increase in the production of neutral aroma-enhancing compounds (NAECs). Notably, the production of the following NAECs including dihydrokiwi lactone (DHKL: 192.86%), 2,4-di-tert-butylphenol (DTBP: 25%), 4-oxoisofolkone (OIFK: 116.66%), 1,9-heptadecadiene-4,6-diyn-3-ol (HDD: 116.67%), β-damastrone (BDS: 116.67%), megastigmatrienone A, B, C and D isomers (MST: 263.36%), 4-hydroxyphenyl retinamide (HOPRA:161.11%), linalool (50%), and benzaldehyde (BA: 66.66%) was increased (Huang et al., 2024b).

The pharmacological activities of these compounds are as follows: DHKL exhibits several biological activities, such as cytotoxic, anti-inflammatory, antimicrobial, anticancer, and antimalarial properties (Shen et al., 2023; Surowiak et al., 2021). DTBP demonstrates diverse bioactivities, including antimicrobial activity, antioxidant properties, anticancer potential, and antibiofilm activity (Kaari et al., 2023; Kavisri et al., 2023). OIFK has been reported with various biological activities, including anticancer, antibacterial, anticonvulsant, antiallergic, anthelmintic, antiviral, antidepressant, analgesic, and antioxidant properties (Siwach and Verma, 2020; Zhu et al., 2020). HDD demonstrated multiple bioactivities, including anticancer, neuroprotection, anti-inflammatory, and antimicrobial (Andersen et al., 2020; Santos et al., 2022). Derivatives of β-damascone have applications in pest management (*Myzus persicae*) and mealworm (*Alphitobius diaperinus*) (Gliszczyńska et al., 2014). MSTA is known for its aroma and flavour properties, but it also exhibits phytotoxic and anti-inflammatory bioactivities (Pan et al., 2019). HOPRA (fenretinide) selectively activates the retinoid receptors and regulates the expression of genes involved in breast cancer and apoptosis (Dmitrovsky, 2004; Sabichi et al., 2003; Zhang et al., 2024). HOPRA is also therapeutically effective against other pathological conditions such as cystic fibrosis, rheumatoid arthritis, acne, and psoriasis (Cazzaniga et al., 2012; Fanjul et al., 1996). Linalool inhibits the growth of pathogens such as *Staphylococcus aureus*, *Escherichia coli*, and *Candida albicans*; suppresses pro-inflammatory cytokine production; relieves pain through modulation of the central nervous system; reduces anxiety and stress while promoting sleep via GABAergic pathways; neutralizes free radicals; prevents neuroinflammation and oxidative stress, offering protection against Alzheimer’s and Parkinson’s diseases; and acts as a natural insecticide and repellent, particularly against mosquitoes and agricultural pest (An et al., 2021; Milanos et al., 2017; Pandur et al., 2024). BA is generally considered safe when used in small concentrations in foods and cosmetics. It inhibits the growth of *Staphylococcus aureus* and *Drosophila melanogaster*, acts as an anti-inflammatory agent, and exhibits anticancer activity through its Schiff bases (Mezgebe and Mulugeta, 2024; Neto et al., 2021; Ullah et al., 2015).

Advancements in metagenomics have greatly accelerated the identification of novel microbial strains. For instance, *Monascus*, *Lactococcus*, and *Aspergillus*, associated with the production of flavour compounds like esters, acids, and methyl ketones in *Monascus*-fermented cheese, were identified through metagenomic analysis (Wang et al., 2024a; Wang et al., 2024b). In one of our earlier studies, we also applied metagenomic analysis, mainly to figure out how different microbes were contributing to NAEC overproduction and nicotine breakdown during the fermentation of RTLC (Huang et al., 2024b). Metatranscriptomic analysis (MTA) is prformed to identify active metabolic pathways, record microorganisms’ responses to environmental change, compare gene activity in different ecological conditions, and attribute the behavior of microbial species to biochemical outcomes (Jovel et al., 2022; Shakya et al., 2019; Singh et al., 2021; Zhang et al., 2024).

The goal of the present study was to perform the MTA to explore the underlying mechanisms behind NAECs production, nicotine breakdown, and improvements in the sensory quality of fermented RTLC by *K. variicola* H8 and the microbial communities.

## 2 Materials and methods

### 2.1 Fermentation of RTLC and GC-MS analysis

In this study, we used our previously characterized *K. variicola* H8 strain for MTA analysis after applying it to RTLC fermentation. Our research group has reported its role in the overproduction of several NAECs, nicotine degradation, and sensory quality improvement. Our previous studies have reported optimized growth conditions for the optimal growth of this strain and a method for quantification of sensory quality (Huang et al., 2024a; Huang et al., 2024b). Since MTA adds another layer to metagenomics analysis for this current study, we decided to stick with the same strain and culture conditions. We collected samples at five different time points, specifically at 0, 8, 16, 24, and 36 h (labeled CK, H8H, H16H, H24H, and H36H, respectively, and took three replicates for each. All samples were then sent to the Shanghai Paisenore Biological Co., Ltd. (China) for metatranscriptomic sequencing.

We also used the same protocol for the extraction, GC-MS analysis, and quantification of NAECs and nicotine in fermented RTLC (Huang et al., 2024b). Statistical significance of correlations was evaluated using Pearson’s test with Benjamini-Hochberg false discovery rate (FDR) correction for multiple testing. Adjusted p-values <0.05 were considered significant.

### 2.2 Metatranscriptome analysis

#### 2.2.1 Metatranscriptome sequencing

Metatranscriptome sequencing in this study was carried out using the Illumina NovaSeq/HiSeq high-throughput platform, which has been widely used in similar microbial transcriptome studies (Bejaoui et al., 2025; Kastanis et al., 2019). We extracted the total mRNA of all microbial species found in the fermented RTLC and then reverse-transcribed it into double-stranded cDNA. Subsequently, cDNA was fragmented, and paired-end libraries were constructed to perform the shotgun sequencing. By doing so, we achieved the broad coverage and high-quality sequencing data (Wang et al., 2009).

#### 2.2.2 Species diversity analysis of transcriptome sequences

To assess microbial diversity and abundance at various fermentation stages, we analyzed the transcriptomic sequences using QIIME2 software (Caporaso et al., 2010). The reference sequences were taxonomically classified using the Lowest Common Ancestor (LCA) algorithm via the Blast2LCA tool (Wang et al., 2022). This allowed us to trace each sequence to its most likely species-level identity. In doing so, we could map out the taxonomic composition of the metatranscriptomic data and retrieve species-level information for each contig (Gautam et al., 2023; Huson et al., 2007).

#### 2.2.3 Functional annotation of transcriptome sequences

For functional insights, we used MMseqs2 to generate a set of non-redundant protein sequences from the transcriptome data (Steinegger and Söding, 2017). These were compared to the carbohydrate-active enzymes (CAZy) database for carbohydrate-active enzyme annotation (Hobbs et al., 2023). Additionally, we annotated gene functions by aligning sequences against several other well-established databases, including KEGG (Mao et al., 2005), UniProt (Camon et al., 2004), and GO (Gene Ontology) (Aleksander et al., 2023) where relevant.

#### 2.2.4 Statistical and visualisation analysis

Statistical analyses were conducted in R, which we also used to visualize differential gene expression patterns in *K. variicola* H8 across the different fermentation time points.

## 3 Results and discussion

### 3.1 Optimal time for the production of NAECs and nicotine degradation in fermented RTLC

In a previous study, we found that fermenting RTLC with *K. variicola* H8 led to a noticeable increase in NAEC production, specifically, 34 compounds were enhanced, making up about 45% of the total. At the same time, nicotine levels dropped by 25%, and sensory quality scores improved by 5.71% (Huang et al., 2024b). However, one concern with prolonged fermentation is the formation of tobacco-specific nitrosamines (TSNAs), which are known carcinogens (Li et al., 2020). Because of that, we wanted to figure out the exact fermentation time to avoid the production of TSNAs.

**FIGURE 1:**
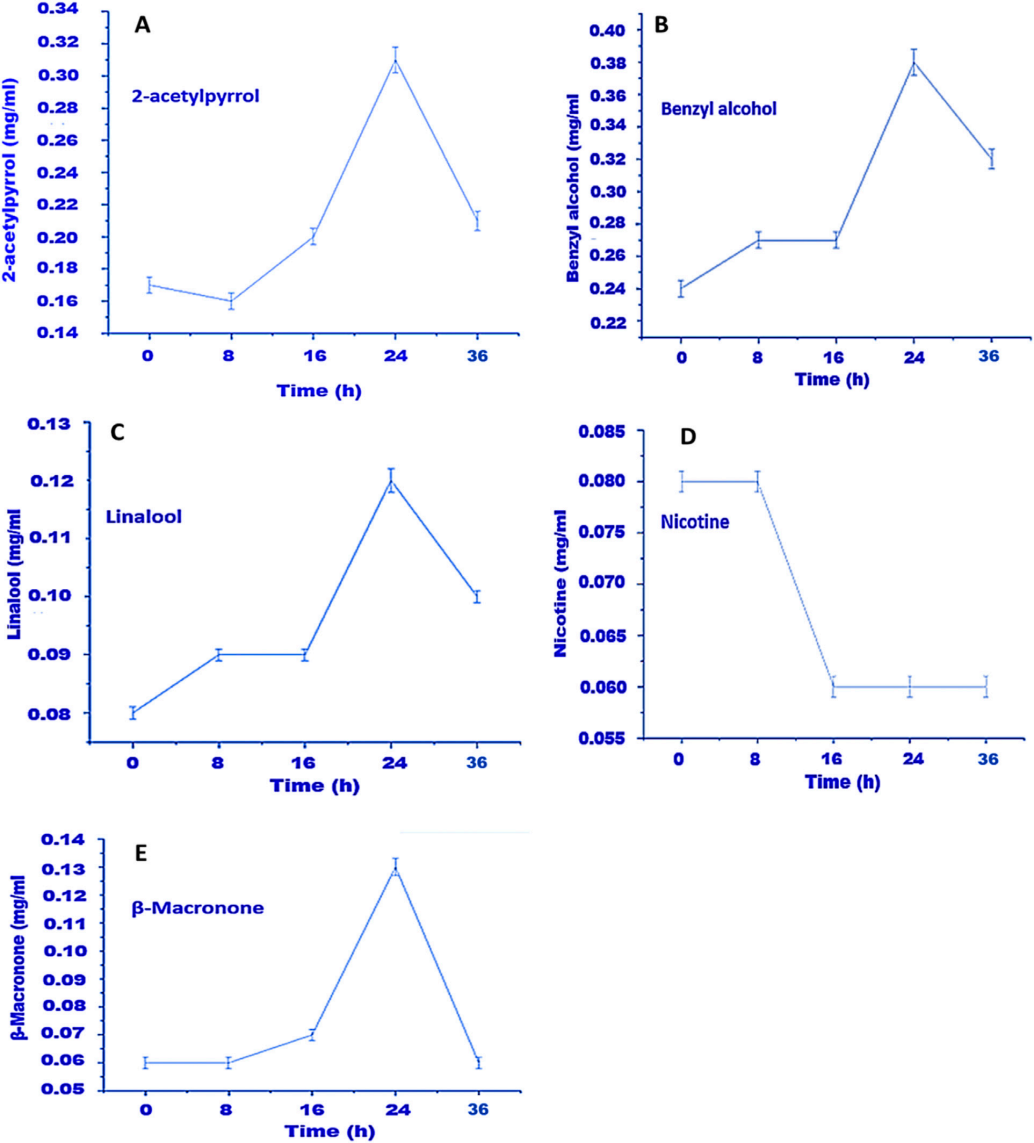
Time-course analysis of NAECs production and nicotine degradation in fermented RTLC. In this study, the time course analysis of 34 NAECs, including nicotine, was performed. Still, temporal variations in the concentrations of only five metabolites, such as **(A)** 2-acetylpyrrol, **(B)** benzyl alcohol, **(C)** linalool, **(D)** nicotine, and **(E)** β-macronone, are presented in this article. The production of **(A)** 2-acetylpyrrol, **(B)** benzyl alcohol, **(C)** linalool, and **(E)** β-macronone was maximum up to the 24th hour of fermentation, while at the same time, the level of nicotine was also decreased in the RTLC **(D)**.

To do this, we ran a time-course analysis, tracking NAEC and nicotine levels over several key points of fermentation. According to the GC-MS results, the most significant rise in NAECs and the most effective reduction in nicotine was observed by the 24-h mark (Figure 1). This spike likely stems from how *K. variicola* H8 utilizes a range of nutrients, including carbohydrates, amino acids, lipids, and even nicotine itself, as fermentation progresses (Ardö, 2006; Liang et al., 2024; Ning et al., 2023; Rodríguez-Bustamante and Sánchez, 2007; Yvon and Rijnen, 2001).

Interestingly, our earlier metagenomic analysis supports this, showing that the RTLC microbiome carries the genetic tools needed to break down those same compounds (Huang et al., 2024b). Other studies have reported similar patterns. For instance, microbes like *Paenarthrobacter nicotinovorans* (Zhang et al., 2022), *Ochrobactrum intermedium* DN2 (Yuan et al., 2006), and *Pseudomonas* sp. Nic22 has all been used to degrade nicotine and enhance tobacco quality (Li et al., 2024). According to Z.-J. Li and colleagues, microbes can use nicotine as a source of both carbon and nitrogen to generate the energy they need for growth (Li et al., 2024).

Based on our findings, the ideal fermentation time when using *K. variicola* H8 appears to be around the 24-h mark. At this point, NAEC production is maximized, nicotine levels are significantly reduced, and harmful TSNAs are not yet a concern (Figure 1). In this study, TSNAs were not quantified, as their formation during fermentation has already been reported in our previous work (Huang et al., 2024b).

### 3.2 Macrotranscriptomics analysis of microbial community dynamics during RTLC fermentation

**FIGURE 2:**
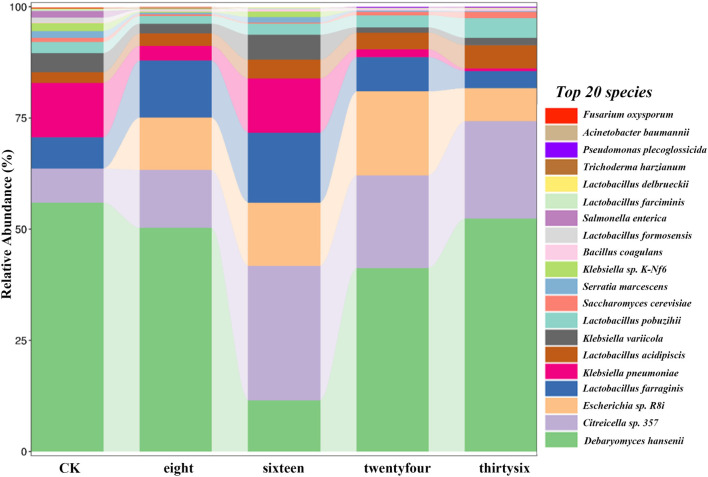
Relative abundance of microbial species is involved in RTLC fermentation. The figure presents the relative abundance of the top 20 dominant microbial species involved in the RTLC fermentation.

**FIGURE 3:**
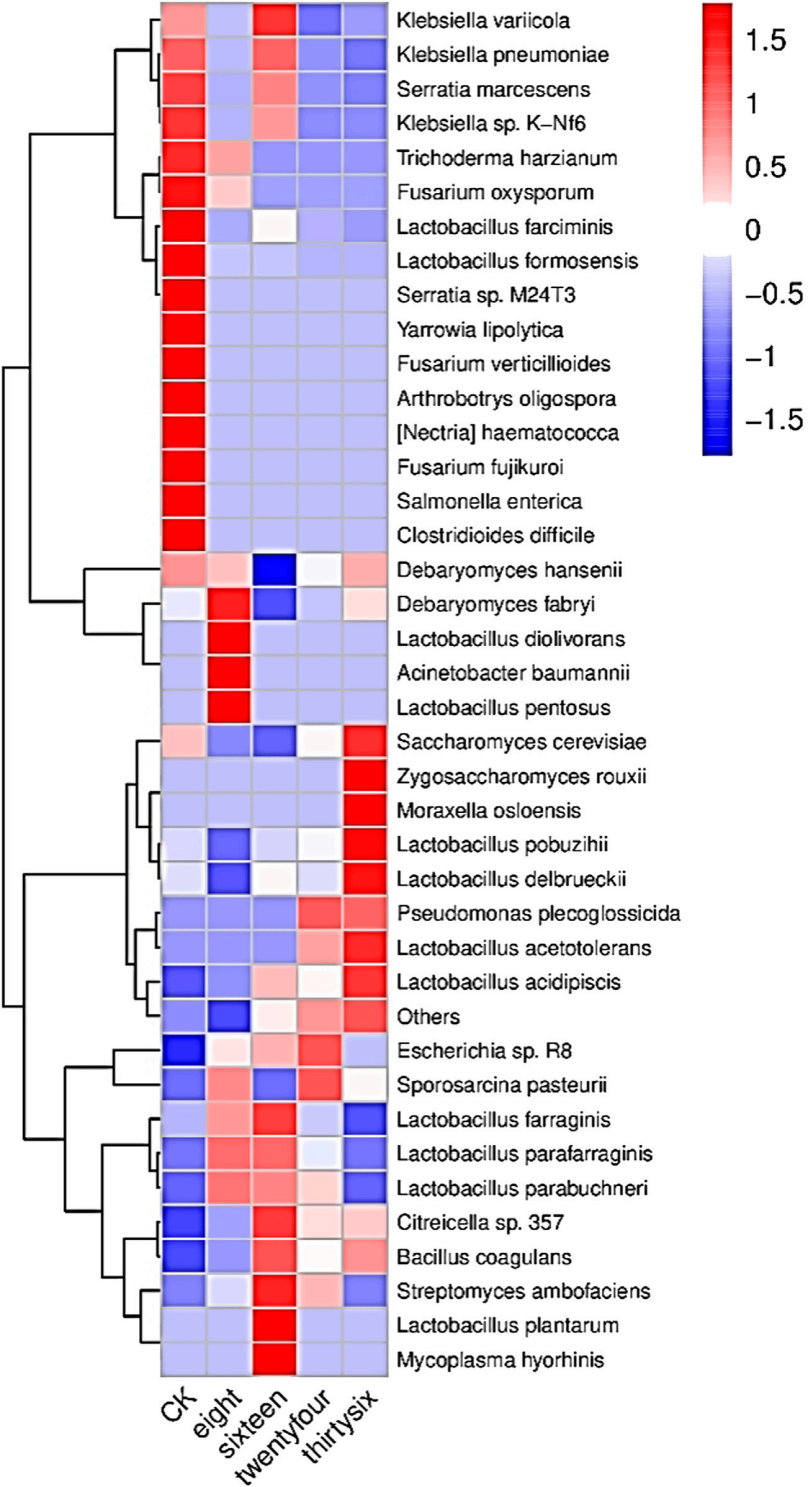
RNA relative abundance of microbial species involved in RTLC fermentation: This heatmap illustrates the transcriptional activity of microbial species involved in RTLC fermentation inoculated with *K. variicola* H8. The color gradient represents RNA abundance, with red indicating high expression and blue indicating low expression. Key species, including *K. variicola*, *K. pneumoniae*, and *Serratia marcescens*, showed the highest activity (16 h) before declining in later stages. The clustering pattern highlights microbial succession, reflecting dynamic interactions influencing fermentation efficiency and flavor development.

The relative abundance of RNA was analyzed to evaluate the development of microbial community structure during the fermentation of RTLC; subsequently, their role in shaping the quality of tobacco products is inferred (Figures 2, 3). Figures 2, 3 present the relative abundance of the top 20 microbial species and the differential gene expression of each organism, respectively, at 0 h, 8 h, 16 h, 24 h, and 36 h of RTLC fermentation. Figure 3 complements the results in Figure 2.

Figure 2 shows *K. variicola* H8, *Bacillus coagulans*, and *Lactobacillus formosensis* as dominant microbial strains. In contrast, Figure 3 indicates that these species, along with *K. variicola*, *Klebsiella pneumoniae*, and *Serratia marcescens*, are transcriptionally active microbial species at 0 h of the fermentation. These species are commonly found in the raw material of plants, and they generally kickstart the fermentation process when they see the opportunity (Suresh, 2023; Hleba et al., 2021; Jia et al., 2008; Rodríguez-Medina et al., 2019).

The results in Figure 2 show that *K. variicola* H8, *Debaryomyces hansenii*, *Citreicella* sp. 357, *Lactobacillus farraginis*, and *Klebsiella pneumoniae* were relatively dominant strains and therefore the relative abundance of their RNA was high (Figure 3) at the beginning of the fermentation (8 h), which indicates that these strains also play a critical role in the fermentation of RTLC. The decomposition of plant material with microbial consortia for the production of high-value compounds has been reported by other studies as well (Gentzke et al., 2022; L. Zou et al., 2024). The cigar fermentation studies have also reported the role of the abovementioned dominant bacterial and yeast species on the production of flavor and metabolic activity (Si et al., 2023; Tao et al., 2024).

It is evident from the results depicted in Figure 2 that the structure of the microbial community involved in the fermentation of RTLC is tending toward equilibrium state in which microbial species adapt to the changing culture environment (16 h). The microbial species such as *Debaryomyces hansenii*, *Lactobacillus farraginis*, and *Citreicella* sp. 357 remain relatively abundant (Figure 2) and transcriptionally active (Figure 3). The relative RNA abundance of *K. variicola* H8 was highest at the 16th hour of RTLC fermentation (Figure 3). In contrast, the relative RNA abundance of early contributors to the RTLC fermentation, such as *Serratia marcescens*, and *K. Pneumoniae*, along with *Citrobacter* sp., *Lactobacillus* sp., and *Aromaticobacter* was also simultaneously decreased (Figure 3) (16 h).

The change in the microbial structure and prevalence of yeast and lactic acid bacterial species has been reported as crucial for RTLC fermentation and the production of flavor compounds in the subsequent stages (Huang et al., 2024b; Pan et al., 2022). The relative species and RNA abundance of *S. cerevisiae* and acid-tolerant lactic acid bacteria like *L. Pobuzihii* increased at 36 h of RTLC fermentation (Figures 2, 3), which explains their role in the carbohydrate metabolism and organic acid production (Tao et al., 2024). On the contrary, the relative species and RNA abundance of *Escherichia coli* decreased (Figures 2, 3), which can be attributed to the following factors, including changes in the pH of the media, depletion of resources, and outnumbered (Li et al., 2020) by *S. cerevisiae* and acid-tolerant lactic acid bacteria like *L. Pobuzihii*.

The results in Figures 2, 3 highlight microbial succession and cooperation, particularly the role of *K. variicola* H8 in initiating RTLC fermentation. This initiation subsequently creates an opportunity for the growth of opportunistic members commonly found in unfermented RTLC. As a result, *K. variicola* H8 strongly influences NAECs production and the overall quality of tobacco products.

### 3.3 Microbial contributions to chemical composition and NAECs dynamics in RTLC fermentation

**FIGURE 4:**
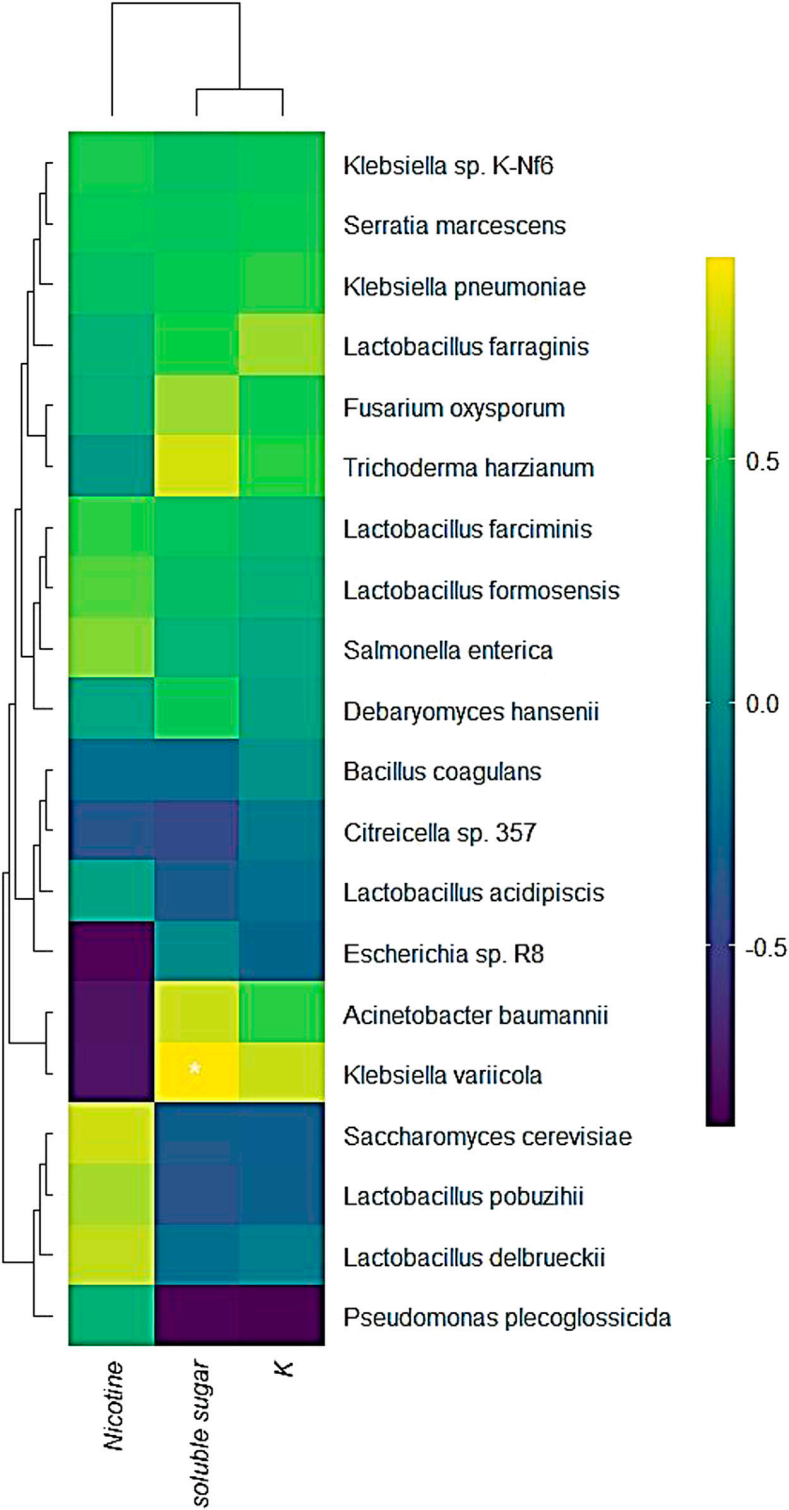
Correlation of microbial transcriptional activity and changes in conventional chemical components during RTLC fermentation. The figure presents a heatmap illustrating the correlation between microbial transcriptional activity and changes in traditional chemical elements, including nicotine, soluble sugar, and potassium (K), during the fermentation of RTLC. The hierarchical clustering reveals distinct microbial associations with these chemical components.

The relationship between the changes in conventional chemical components and microbial transcription after RTLC fermentation was evaluated (Figure 4). The correlation analysis reveals a strong association between microbial activity and variations in nicotine, soluble sugar, and potassium (K) content. *K. variicola* exhibited a significant positive correlation with soluble sugar, indicating its potential role in sugar metabolism during fermentation. This aligns with previous studies suggesting that *Klebsiella* species actively participate in carbohydrate metabolism and contribute to the breakdown of complex sugars in fermentation systems (Duran-Bedolla et al., 2021).

Furthermore, *Escherichia* sp. R8 and *Acinetobacter baumannii* showed a negative correlation with nicotine content, suggesting their potential involvement in nicotine degradation (Figure 4). Similar findings have been reported in studies where *Acinetobacter* and *Pseudomonas* species possess nicotine-catabolizing enzymes, contributing to their biotransformation during microbial fermentation (Wang et al., 2007). The weak correlation of *K. variicola* with nicotine suggests that its primary metabolic activity is centered around sugar utilization rather than nicotine degradation (Figure 4) (Huang et al., 2024b). Interestingly, the correlation analysis also revealed that *K. variicola* exhibited a negative correlation with potassium content (Figure 4). The underlying mechanism remains unclear; however, previous research suggests that potassium plays a crucial role in bacterial osmoregulation, stress responses, and metabolic activity, which could indirectly influence microbial interactions in the fermentation system (Stautz et al., 2021).

Additionally, *Lactobacillus acidipiscis* demonstrated a positive correlation with soluble sugar content, reinforcing its known role in lactic acid fermentation and carbohydrate metabolism (Figure 4). This is consistent with studies showing that *Lactobacillus* species are key players in sugar fermentation and organic acid production (Cufaoglu and Erdinc, 2023; Hedberg et al., 2008; Stautz et al., 2021).

The findings highlight the complex interactions between microbial communities and chemical composition changes during fermentation. *K. variicola* is a dominant sugar-fermenting bacterium, whereas *Acinetobacter* and *Escherichia* contribute to nicotine degradation.

**FIGURE 5:**
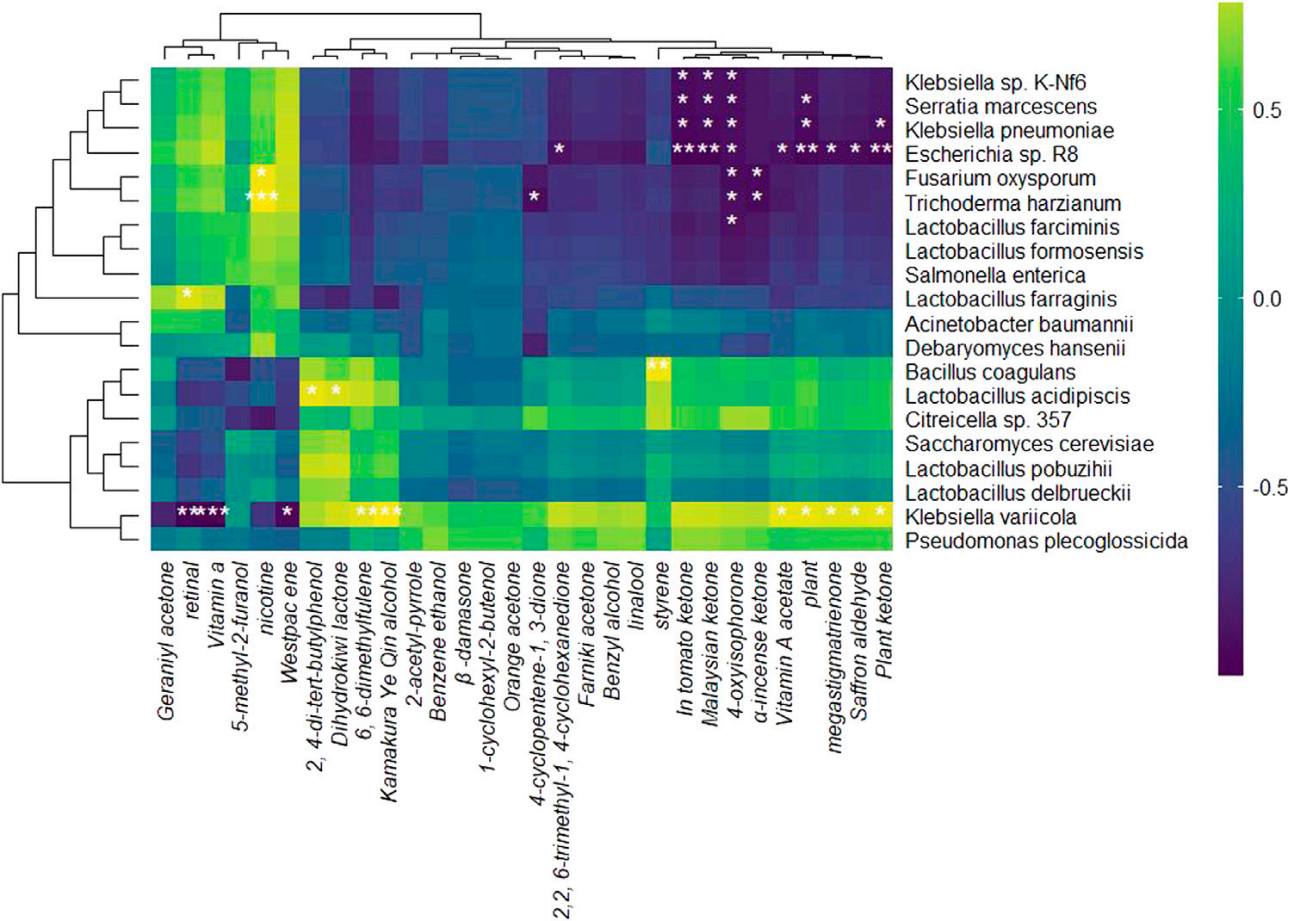
Heatmap of correlation analysis between microbial species and NAECs production. The green indicates a positive correlation, blue represents neutral relationships, and purple signifies a negative correlation. Significant correlations are marked with asterisks.

The correlation analysis between relative RNA abundance and NAECs during the RTLC fermentation reveals significant relationships between specific microbes and volatile aroma compounds (Figure 5). The results in Figure 5 demonstrate a strong positive correlation between *K. variicola* H8 and key NAECs (2,4-Di-tert-butylphenol, dihydroactinidiolide, phenylethyl alcohol, benzyl alcohol, linalool, β-damascenone, 1-cyclohexyl-2-butenol, nerylacetone, solanone, α-cyperone, farnesyl acetone, and 2-acetyl-1H-pyrrole). Different studies have reported that these NAECs add aroma to tobacco products, such as β-damascenone and linalool, which add floral and fruity flavor (Gong et al., 2023; Pan et al., 2022). In addition, significant positive correlation between *K. variicola* H8 and compounds like phytol, phytone, megastigmatrienone, and saffron aldehyde was observed which indicates that this strain plays a vital role in the biosynthesis of these compounds (Figure 5). These findings align with previous studies that demonstrate how *Klebsiella* species contribute to the transformation of precursor molecules into aromatic volatiles (Chen et al., 2021; Huang et al., 2024b).

The correlation analysis of microbial species and aroma compounds exhibited that the Westpac and vitamin A are negatively correlated with *K. variicola* H8 (Figure 5), which indicates that both compounds are either used by these organisms for their growth or their production was inhibited during the RTLC fermentation. Similar outcomes have been reported by other studies, which state that fermentations with microbial organisms result in reduced concentrations of Westpac and vitamin A (Denter et al., 1998; Whited et al., 2002). The aroma characteristics and sensory attributes of 2,4-di-tert-butylphenol and dihydrokiwifolactone are well-registered in the tobacco and food industry (Wang et al., 2023). In this, we also discovered a positive correlation between these compounds and *L. acidipiscis* (Figure 5).

The correlation analysis between microbial species and NAECs production demonstrates that *K. variicola* H8 and other microbial species play a critical role in enriching the RTLC with NAECs.

### 3.4 CAZy enzyme dynamics and their role in carbohydrate degradation during RTLC fermentation

**FIGURE 6:**
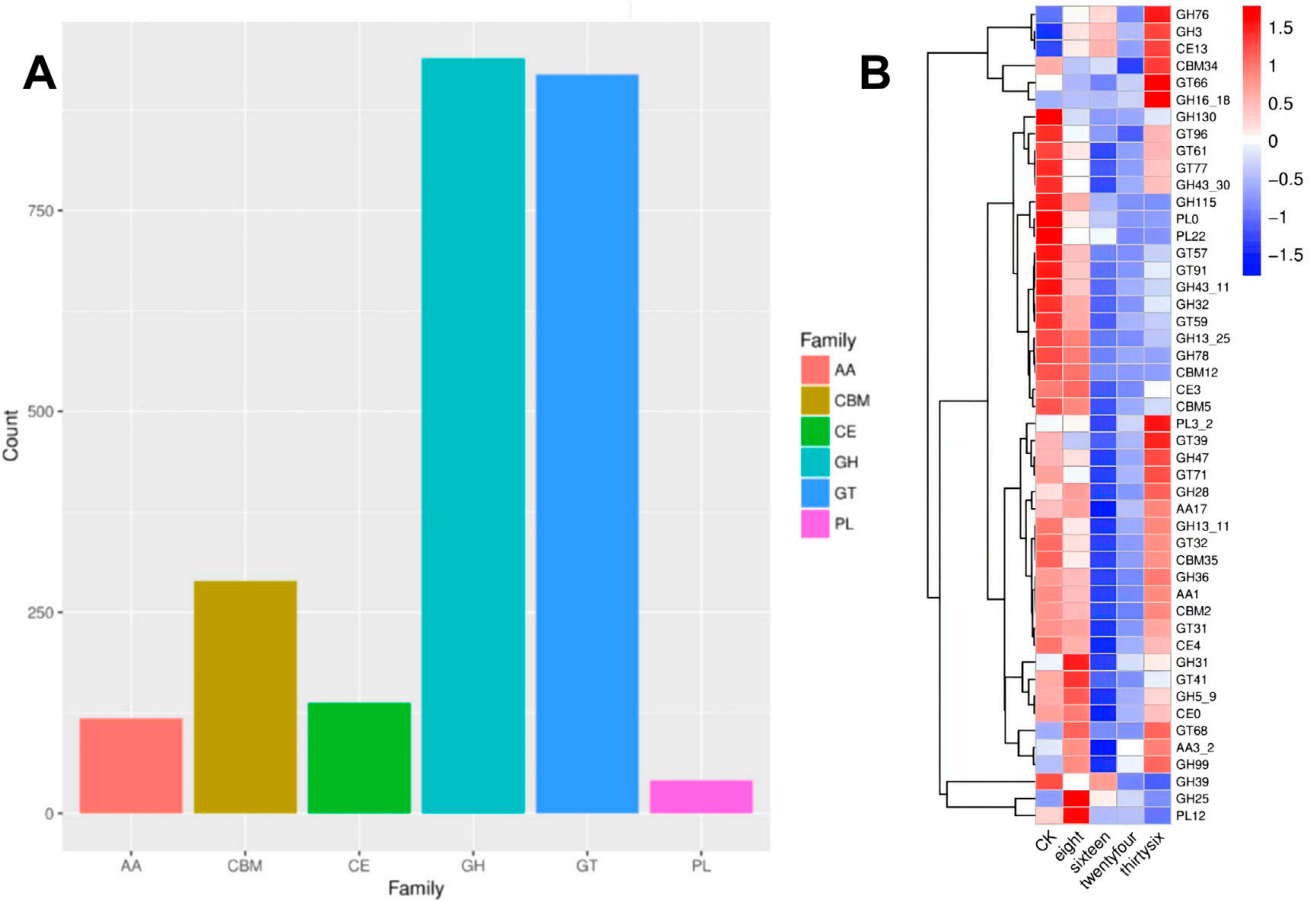
**(A)** Transcript abundance of CAZy enzymes in K. variicola H8 during RTLC Fermentation. The bar chart presents the distribution of CAZy family transcripts in *K. variicola* H8 during the fermentation of RTLC. Among the enzyme families analyzed, glycoside hydrolases (GH) and glycosyltransferases (GT) exhibit the highest transcript abundance, with counts exceeding 900. Carbohydrate-binding modules (CBM) and carbohydrate esterases (CE) show moderate transcript levels, while auxiliary activity (AA) enzymes and polysaccharide lyases (PL) have the lowest representation. **(B)** Heatmap of glycoside hydrolase gene expression during fermentation. This figure is a heatmap displaying the transcriptional changes of various glycoside hydrolase (GH) family genes during fermentation at different times (CK, 8 h, 16 h, 24 h, and 36 h). The color scale represents relative expression levels, with red indicating upregulation and blue indicating downregulation. The hierarchical clustering on the left group genes with similar expression patterns shows that several GH genes exhibit increased transcriptional activity as fermentation progresses.

The transcriptional analysis of flavor-enhancing bacteria during the fermentation of RTLC was performed using the CAZy enzyme family annotation (Lombard et al., 2014). The distribution of CAZy-related transcripts is shown in Figure 6A. Among the enzyme families, glycoside hydrolases (GH) exhibited the highest transcriptional abundance (955 transcripts), followed closely by glycosyltransferases (GT) (931 transcripts). Polysaccharide lyases (PL) were the least represented (42 transcripts).

Other enzyme families, such as carbohydrate esterases (CE), auxiliary activity (AA), and carbohydrate-binding modules (CBMs), which are associated with carbohydrate metabolism, also played a vital role in the RTLC fermentation, and their contribution was recorded in terms of 137, 118, and 293 transcripts, respectively (Figure 6A). The presence of CBM transcripts highlights their role in the hydrolysis of carbohydrate, which involves the facilitation of the enzyme-substrate interactions (Boraston et al., 2004). The presence of high levels of GH and GT transcripts indicates that carbohydrate molecules in the RTLC were mainly degraded through hydrolysis and glycosylation reactions (Chen et al., 2025; Muradova et al., 2023; Pan et al., 2022; Parapouli et al., 2019). In addition, the difference in the relative abundance of GH, GT, PL, CE, AA, and CBM in this study (Figure 6A) endorses the findings of previously published studies (Cantarel et al., 2009; Lombard et al., 2014). Furthermore, these studies have also reported that GH, GT, PL, CE, AA, and CBM catalyze the breakdown of carbohydrates into NAECs (Cantarel et al., 2009; Lombard et al., 2014).

The change in the transcriptional profile of GH, GT, PL, CE, AA, and CBM over time is presented in Figure 6B, which shows that the expression level of GH was severalfold increased (Figure 6B). Among these, GH130 exhibited a striking 4.2-fold increase, followed by GH43-30 (3.7-fold), GH78 (2.5-fold), and GH31 (3.8-fold) by 36 h. These results suggest that these glycoside hydrolases are crucial in carbohydrate degradation and aroma formation. GH47, GH32, GH39, GH76, GH3, GH13-25, and GH28 also displayed transcriptional upregulation, ranging from 1.6 to 4.5-fold. Interestingly, these observations align with findings from other fermentation studies. For instance, research on *Debaryomyces hansenii* Y4 during Sichuan South-road Dark Tea fermentation identified the upregulation of GH families such as GH17, GH18, GH76, GH31, GH47, and GH2, where enzymes like β-galactosidase and mannosidase influenced the tea’s flavour by degrading polysaccharides and oligosaccharides (Zou et al., 2023). Similarly, comparative genomics of lactic acid bacteria emphasized the genetic basis for flavour compound biosynthesis, including the role of GHs in forming flavour-active metabolites (Liu et al., 2008).

The findings on the expression patterns of the GH gene suggest that enhancing the expression of specific glycoside hydrolases can improve the efficiency of the production of NAECs and improve the flavor quality of the product. Therefore, this approach can be applied to the food and tobacco industries, where controlled microbial fermentation could be optimized to enhance sensory attributes.

### 3.5 Correlation of glycoside hydrolase expression with NAEC production in RTLC fermentation

**FIGURE 7:**
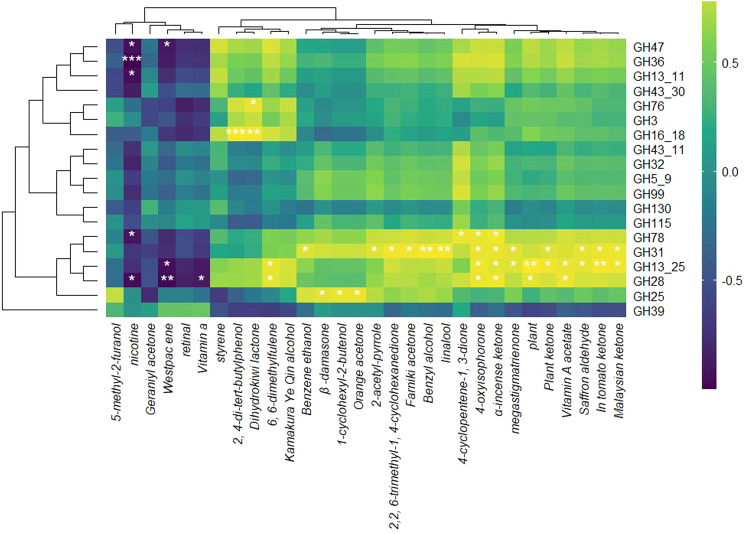
Correlation between NAECs compounds and expression of GH gene. This heatmap illustrates the Pearson correlation coefficients between the expression levels of GH family genes and the concentrations of NAECs during *K. variicola* H8’s fermentation of RTLC. The rows represent different GH family genes, while the columns denote individual aroma compounds detected in the fermented RTLC. The color gradient from purple to yellow reflects the strength and direction of correlation, with purple indicating strong negative correlations (≤−0.5), yellow indicating strong positive correlations (≥0.5), and green denoting weak or no correlation (around 0). Asterisks (*) represent statistically significant correlations (*p* < 0.05).

A correlation analysis was performed to investigate the relationship between GH family transcript abundance and the production of NAECs during the fermentation of RTLC by *K. variicola* H8. The resulting heatmap (Figure 7) illustrates positive and negative associations between specific GH families and various NAECs, underscoring the critical role of microbial enzymatic activity in modulating tobacco aroma profiles.

Several GH families, particularly GH78, GH13_25, GH31, GH28, GH16_18, and GH76, exhibited strong positive correlations with a broad range of aroma compounds (Figure 7). These findings suggest that the overexpression of these enzymes during fermentation may facilitate the enzymatic release of volatile compounds from glycosidically bound precursors, a mechanism widely supported in the literature (Hu et al., 2016a; Hu et al., 2016b). Among the aroma compounds, megastigmatrienone, an essential contributor to the sweet and woody aroma, showed exceptionally high positive correlations with GH78 and GH13_25 (Figure 7), consistent with reports that it is released via microbial deglycosylation of carotenoid-derived precursors (Hu et al., 2016a; Hu et al., 2016b). Similarly, the fruity aroma compound dihydrokiwi lactone is strongly associated with GH31, GH28, and GH16_18 (Figure 7), further supporting their involvement in lactone biotransformation. This aligns with prior findings where microbial strains like *Yarrowia lipolytica* converted hydroxy fatty acids into aroma-active lactones such as γ-decalactone through enzymatic processes (AL Mualad et al., 2022; Silva et al., 2021).

Additional volatiles such as benzyl alcohol, farnesyl acetone, and linalool, known for their floral and woody aromatic profiles, were also positively associated with GH31 and GH28 (Figure 7). Previous research has shown that these compounds often occur in plants as glycosidically bound forms, which GH enzymes can hydrolyze to release the free aroma-active compounds (Sarry and Günata, 2004; Zheng et al., 2019). While direct evidence for GH-mediated release of farnesyl acetone remains limited, the general role of glycoside hydrolases in liberating bound volatiles supports this hypothesis. The antioxidant compound 2,4-di-tert-butylphenol, which contributes woody notes, also showed positive correlations with GH16_18 and GH76 (Figure 7), consistent with their potential role in producing phenolic volatiles (Leonard et al., 2021).

Other key NAECs, such as 1-cyclohexyl-2-butanone, orange peptone, 4-cyclopentene-3-one, and 6-methyl-5-hepten-2-one, exhibited significant positive correlations with GH31, GH78, GH13_25, and GH115 (Figure 7). These compounds produce sweet, citrus, smoky, and fruity sensory qualities in the tobacco products (Maldonado-Robledo et al., 2003; Yan et al., 2022). Likewise, kamakui yeoh alcohol displayed a positive correlation with GH31 and GH16_18 (Figure 7), highlighting the broad substrate specificity of these enzymes (Lindsay et al., 2022).

The GH31 demonstrated a negative correlation with Westpacene and vitamin A (Figure 7). A similar trend has also been observed between these compounds and *K. variicola* H8 in Figure 5. Therefore, these results confirm the degradation of Westpacene/vitamin A through hydrolysis during the RTLC fermentation, which is perhaps for microbial growth or NAECs overproduction or both. Other studies have associated GH31 activity with terpene metabolism and glycoside conversion (Caffall and Mohnen, 2009; Cai et al., 2023). Additionally, further transcriptomic correlations revealed that GH78, GH13_25, GH28, and GH31 were also associated with increases in secondary aroma compounds such as kauri ketones, α-hydroxybenzoin, lycopene, and macadamia trienone.

The above results unveil the central role of GH, particularly GH78, GH13_25, GH31, GH28, GH16_18, and GH76, in producing NAECs during the fermentation of RTLC.

## 4 Conclusion

This study employed high-throughput metatranscriptomic sequencing to explore the microbial activity and functional gene expression during the fermentation of reconstituted tobacco leaf concentrate (RTLC). The results indicated a significant increase in the relative transcript abundance of *K. variicola* H8, *Citrobacter*, and *Lactobacillus* during fermentation. By 16 h, the transcriptional activity across dominant microbial taxa reached a relatively balanced state, suggesting a transient equilibrium in the microbial community. Functional gene expression analysis further highlighted a strong positive correlation between *K. variicola* H8 transcript levels and changes in water-soluble sugar content, with weaker correlations observed for nitrogen and potassium dynamics. Significantly, inoculation with aroma-enhancing microbes stimulated the upregulation of key metabolic pathways involved in glycan biosynthesis, lipid metabolism, terpenoid and polyketide synthesis, and amino acid metabolism, particularly phenylalanine. A suite of glycoside hydrolases (GH), including GH76, GH3, GH13, GH28, GH31, GH99, GH25, and GH78, was identified as central players in the release of aroma-active compounds, likely contributing to the improvement in sensory quality. However, by 36 h of fermentation, increased expression of stress-related functions, including apoptosis, was observed. This, combined with sensory evaluation and chemical analysis, indicated a decline in RTLC quality, suggesting a critical threshold for optimal fermentation duration.

These findings deepen our understanding of the metabolic and microbial dynamics driving aroma compound production during tobacco fermentation. They also highlight *K. variicola* H8 as a key functional bacterium in shaping the chemical and sensory profiles of fermented RTLC, offering a promising avenue for improving tobacco product quality through targeted microbial interventions.

## Data Availability

The datasets generated and/or analyzed during the current study are available in the Genome Sequence Archive (Genomics, Proteomics and Bioinformatics 2021) in the National Genomics Data Center (Nucleic Acids Res 2022), China National Center for Bioin formation/Beijing Institute of Genomics, Chinese Academy of Sciences (GSA:CRA010523; https://bigd.big.ac.cn/gsa/browse/CRA010523, accessed on 5 April 2023).

## References

[B1] Al Mualad W. N. A. Bouchedja D. N. Selmania A. Maadadi R. Ikhlef A. Kabouche Z. (2022). Yeast Yarrowia lipolytica as a biofactory for the production of lactone-type aroma gamma-decalactone using castor oil as substrate. Chem. Pap. 76 (12), 7715–7728. 10.1007/s11696-022-02435-2

[B2] Aleksander S. A. Balhoff J. Carbon S. Cherry J. M. Drabkin H. J. Ebert D. (2023). The gene ontology knowledgebase in 2023. Genetics 224 (1), iyad031. 10.1093/genetics/iyad031 36866529 PMC10158837

[B3] An Q. Ren J. N. Li X. Fan G. Qu S. S. Song Y. (2021). Recent updates on bioactive properties of linalool. Food Funct. 12 (21), 10370–10389. 10.1039/d1fo02120f 34611674

[B4] Andersen C. B. Runge Walther A. Pipó-Ollé E. Notabi M. K. Juul S. Eriksen M. H. (2020). Falcarindiol purified from carrots leads to elevated levels of lipid droplets and upregulation of peroxisome proliferator-activated Receptor-γ gene expression in cellular models. Front. Pharmacol. 11, 565524. 10.3389/fphar.2020.565524 32982759 PMC7485416

[B5] Ardö Y. (2006). Flavour formation by amino acid catabolism. Biotechnol. Adv. 24 (2), 238–242. 10.1016/j.biotechadv.2005.11.005 16406465

[B6] Banožić M. Babić J. Jokić S. (2020). Recent advances in extraction of bioactive compounds from tobacco industrial waste-a review. Industrial Crops Prod. 144, 112009. 10.1016/j.indcrop.2019.112009

[B7] Bejaoui S. Nielsen S. H. Rasmussen A. Coia J. E. Andersen D. T. Pedersen T. B. (2025). Comparison of illumina and Oxford nanopore sequencing data quality for Clostridioides difficile genome analysis and their application for epidemiological surveillance. BMC Genomics 26 (1), 92. 10.1186/s12864-025-11267-9 39885402 PMC11783910

[B8] Boraston A. B. Bolam D. N. Gilbert H. J. Davies G. J. (2004). Carbohydrate-binding modules: fine-tuning polysaccharide recognition. Biochem. J. 382 (3), 769–781. 10.1042/BJ20040892 15214846 PMC1133952

[B9] Caffall K. H. Mohnen D. (2009). The structure, function, and biosynthesis of plant cell wall pectic polysaccharides. Carbohydr. Res. 344 (14), 1879–1900. 10.1016/j.carres.2009.05.021 19616198

[B10] Cai Z. Wei Y. Shi A. Zhong J. Rao P. Wang Q. (2023). Correlation between interfacial layer properties and physical stability of food emulsions: current trends, challenges, strategies, and further perspectives. Adv. Colloid Interface Sci. 313, 102863. 10.1016/j.cis.2023.102863 36868168

[B11] Camon E. Magrane M. Barrell D. Lee V. Dimmer E. Maslen J. (2004). The gene ontology annotation (GOA) database: sharing knowledge in uniprot with gene ontology. Nucleic Acids Res. 32 (DATABASE ISS), 262D–266. 10.1093/nar/gkh021 14681408 PMC308756

[B12] Cantarel B. I. Coutinho P. M. Rancurel C. Bernard T. Lombard V. Henrissat B. (2009). The carbohydrate-active EnZymes database (CAZy): an expert resource for glycogenomics. Nucleic Acids Res. 37 (Suppl. 1), D233–D238. 10.1093/nar/gkn663 18838391 PMC2686590

[B13] Caporaso J. G. Kuczynski J. Stombaugh J. Bittinger K. Bushman F. D. Costello E. K. (2010). QIIME allows analysis of high-throughput community sequencing data. Nat. Methods 7 (5), 335–336. 10.1038/nmeth.f.303 20383131 PMC3156573

[B14] Cazzaniga M. Varricchio C. Montefrancesco C. Feroce I. Guerrieri-Gonzaga A. (2012). Fenretinide (4-HPR): a preventive chance for women at genetic and familial risk? J. Biomed. Biotechnol. 2012, 1–9. 10.1155/2012/172897 22500077 PMC3303873

[B15] Chen L. Zhang L. L. Ren J. N. Li X. Fan G. Pan S. Y. (2021). Screening a strain of klebsiella sp. O852 and the optimization of fermentation conditions for trans-dihydrocarvone production. Molecules 26 (9), 2432. 10.3390/molecules26092432 33922023 PMC8122266

[B16] Chen Z. Song Y. Yan Y. Chen W. Ren T. Ma A. (2025). Characterization of an epilactose-producing cellobiose 2-epimerase from clostridium sp. TW13 and reutilization of waste milk. Food Chem. 480, 143948. 10.1016/J.FOODCHEM.2025.143948 40138832

[B17] Cufaoglu G. Erdinc A. N. (2023). An alternative source of probiotics: water kefir. Food Front. 4 (1), 21–31. 10.1002/fft2.200

[B18] Denter J. Rehm H. J. Bisping B. (1998). Changes in the contents of fat-soluble vitamins and provitamins during Tempe fermentation. Int. J. Food Microbiol. 45 (2), 129–134. 10.1016/S0168-1605(98)00155-X 9924943

[B19] Dmitrovsky E. (2004). Fenretinide activates a distinct apoptotic pathway. J. Natl. Cancer Inst. 96 (17), 1264–1265. 10.1093/jnci/djh268 15339958

[B20] Duran-Bedolla J. Garza-Ramos U. Rodríguez-Medina N. Aguilar Vera A. Barrios-Camacho H. (2021). Exploring the environmental traits and applications of Klebsiella variicola. Braz. J. Microbiol. 52 (4), 2233–2245. 10.1007/s42770-021-00630-z 34626346 PMC8578232

[B21] Fanjul A. N. Delia D. Pierotti M. A. Rideout D. Qiu J. Pfahl M. (1996). 4-Hydroxyphenyl retinamide is a highly selective activator of retinoid receptors. J. Biol. Chem. 271 (37), 22441–22446. 10.1074/jbc.271.37.22441 8798408

[B22] Gautam A. Zeng W. Huson D. H. (2023). MeganServer: facilitating interactive access to metagenomic data on a server. Bioinformatics 39 (3), btad105. 10.1093/bioinformatics/btad105 36825821 PMC9991050

[B23] Gentzke A. S. Wang T. W. Cornelius M. Park-Lee E. Ren C. Sawdey M. D. (2022). Tobacco product use and associated factors among middle and high school students - national youth tobacco survey, United States, 2021. Morb. Mortal. Wkly. Rep. Surveillance Summ. 71 (5), 1–29. 10.15585/mmwr.ss7105a1 35271557 PMC8923300

[B24] Gliszczyńska A. Dancewicz K. Hnatejko M. Szczepanik M. Gabryś B. (2014). Synthesis of β-damascone derivatives with a lactone ring and their feeding deterrent activity against aphids and lesser mealworms. RSC Adv. 4 (74), 39248–39256. 10.1039/c4ra03939d

[B25] Gong Y. Li J. Deng X. Chen Y. Chen S. Huang H. (2023). Application of starch degrading bacteria from tobacco leaves in improving the flavor of flue-cured tobacco. Front. Microbiol. 14, 1211936. 10.3389/fmicb.2023.1211936 37440887 PMC10335769

[B26] Hadj Saadoun J. Bertani G. Levante A. Vezzosi F. Ricci A. Bernini V. (2021). Fermentation of agri-food waste: a promising route for the production of aroma compounds. Foods 10 (4), 707. 10.3390/foods10040707 33810435 PMC8066995

[B27] Hedberg M. Hasslöf P. Sjöström I. Twetman S. Stecksén-Blicks C. (2008). Sugar fermentation in probiotic bacteria - an *in vitro* study. Oral Microbiol. Immunol. 23 (6), 482–485. 10.1111/j.1399-302X.2008.00457.x 18954354

[B28] Hleba L. Hlebová M. Kováčik A. Čuboň J. Medo J. (2021). Carbapenemase producing klebsiella pneumoniae (Kpc): what is the best maldi-tof ms detection method. Antibiotics 10 (12), 1549. 10.3390/antibiotics10121549 34943761 PMC8698427

[B29] Hobbs E. E. M. Gloster T. M. Pritchard L. (2023). Cazy_webscraper: local compilation and interrogation of comprehensive CAZyme datasets. Microb. Genomics 9 (8), mgen001086. 10.1099/mgen.0.001086 37578822 PMC10483417

[B30] Hu K. Qin Y. Tao Y. S. Zhu X. L. Peng C. T. Ullah N. (2016a). Potential of glycosidase from non-saccharomyces isolates for enhancement of wine aroma. J. Food Sci. 81 (4), M935–M943. 10.1111/1750-3841.13253 26954887

[B31] Hu K. Zhu X. L. Mu H. Ma Y. Ullah N. Tao Y. S. (2016b). A novel extracellular glycosidase activity from Rhodotorula mucilaginosa: its application potential in wine aroma enhancement. Lett. Appl. Microbiol. 62 (2), 169–176. 10.1111/lam.12527 26606736

[B32] Huang S. Zhang L. Yan M. Yang J. Rasool A. Manzoor R. (2024a). Growth characteristics of aroma-enhancing bacteria in reconstituted tobacco extracts using isothermal microcalorimetry. Pak. J. Bot. 56 (4). 10.30848/PJB2024-4

[B33] Huang S. Zhu L. Wang K. Zhang X. Mao D. Rasool A. (2024b). Unravel the supremacy of Klebsiella variicola over native microbial strains for aroma-enhancing compound production in reconstituted tobacco concentrate through metagenomic analysis. Metabolites 14 (3), 158. 10.3390/metabo14030158 38535318 PMC10971923

[B34] Huson D. H. Auch A. F. Qi J. Schuster S. C. (2007). MEGAN analysis of metagenomic data. Genome Res. 17 (3), 377–386. 10.1101/gr.5969107 17255551 PMC1800929

[B35] Jia C. C. Hong W. Z. Zhang R. Chen G. X. (2008). Emergence of *Serratia marcescens*, *Klebsiella pneumoniae*, and *Escherichia coli* isolates possessing the plasmid-mediated carbapenem-hydrolyzing β-lactamase KPC-2 in intensive care units of a chinese hospital. Antimicrob. Agents Chemother. 52 (6), 2014–2018. 10.1128/AAC.01539-07 18332176 PMC2415814

[B36] Jovel J. Nimaga A. Jordan T. O’Keefe S. Patterson J. Thiesen A. (2022). Metagenomics Versus metatranscriptomics of the murine gut microbiome for assessing microbial metabolism during inflammation. Front. Microbiol. 13, 829378. 10.3389/fmicb.2022.829378 35185850 PMC8851394

[B37] Kaari M. Joseph J. Manikkam R. Kalyanasundaram R. Sivaraj A. Anbalmani S. (2023). A novel finding: 2,4-Di-tert-butylphenol from Streptomyces bacillaris ANS2 effective against *Mycobacterium tuberculosis* and cancer cell lines. Appl. Biochem. Biotechnol. 195 (11), 6572–6585. 10.1007/s12010-023-04403-2 36881320

[B38] Kastanis G. J. Santana-Quintero L. V. Sanchez-Leon M. Lomonaco S. Brown E. W. Allard M. W. (2019). In-depth comparative analysis of Illumina ® MiSeq run metrics: development of a wet-lab quality assessment tool. Mol. Ecol. Resour. 19 (2), 377–387. 10.1111/1755-0998.12973 30506954 PMC6487961

[B39] Kavisri M. Malathy B. R. Lavanya G. Seema S. Jemmy Christy H. Alex Anand D. (2023). Molecular structure and bioactivities of 2, 4-Ditert butyl phenol extracted from Plumbago zeylanica, investigated using HPLC and NMR. Biomass Convers. Biorefinery 14, 23793–23803. 10.1007/s13399-023-04514-0

[B40] Leonard W. Zhang P. Ying D. Adhikari B. Fang Z. (2021). Fermentation transforms the phenolic profiles and bioactivities of plant-based foods. Biotechnol. Adv. 49, 107763. 10.1016/j.biotechadv.2021.107763 33961978

[B41] Li J. Zhao Y. Qin Y. Shi H. (2020). Influence of microbiota and metabolites on the quality of tobacco during fermentation. BMC Microbiol. 20 (1), 356. 10.1186/s12866-020-02035-8 33213368 PMC7678276

[B42] Li Z.-J. Yang D.-D. Wei Z.-Y. Huang J. Chi Y.-Q. Lu Y.-X. (2024). Reduction of nicotine content in tobacco through microbial degradation: research progress and potential applications. Biotechnol. Biofuels Bioprod. 17 (1), 144. 10.1186/s13068-024-02593-3 39695820 PMC11656995

[B43] Liang B. Bai X. Wang Y. Li X. Kong Y. Li X. (2024). Effect of five lactic acid bacteria on the flavor quality of fermented sweet potato juice. Food Chem. X 24, 102023. 10.1016/J.FOCHX.2024.102023 39655217 PMC11626060

[B44] Lindsay M. A. Granucci N. Greenwood D. R. Villas-Boas S. G. (2022). Identification of new natural sources of flavour and aroma metabolites from solid-state fermentation of agro-industrial By-Products. Metabolites 12 (2), 157. 10.3390/metabo12020157 35208231 PMC8877680

[B45] Liu M. Nauta A. Francke C. Siezen R. J. (2008). Comparative genomics of enzymes in flavor-forming pathways from amino acids in lactic acid bacteria. Appl. Environ. Microbiol. 74 (15), 4590–4600. 10.1128/AEM.00150-08 18539796 PMC2519355

[B46] Lombard V. Golaconda Ramulu H. Drula E. Coutinho P. M. Henrissat B. (2014). The carbohydrate-active enzymes database (CAZy) in 2013. Nucleic Acids Res. 42 (D1), D490–D495. 10.1093/nar/gkt1178 24270786 PMC3965031

[B47] Ma Y. Wei Z. Xiao X. Yu K. Huang H. Tan J. (2024). Investigating the impact of various sorghum types on the key aroma compounds of Sichuan Xiaoqu Baijiu through application of the sensomics approach. Food Chem. X 22, 101367. 10.1016/J.FOCHX.2024.101367 38756476 PMC11096981

[B48] Maldonado-Robledo G. Rodriguez-Bustamante E. Sanchez-Contreras A. Rodriguez-Sanoja R. Sanchez S. (2003). Production of tobacco aroma from lutein. Specific role of the microorganisms involved in the process. Appl. Microbiol. Biotechnol. 62 (5–6), 484–488. 10.1007/s00253-003-1315-6 12827317

[B49] Mao X. Cai T. Olyarchuk J. G. Wei L. (2005). Automated genome annotation and pathway identification using the KEGG orthology (KO) as a controlled vocabulary. Bioinformatics 21 (19), 3787–3793. 10.1093/bioinformatics/bti430 15817693

[B50] Mezgebe K. Mulugeta E. (2024). Synthesis and pharmacological activities of schiff bases with some transition metal complexes: a review. Med. Chem. Res. 33 (3), 439–463. 10.1007/s00044-024-03192-5

[B51] Milanos S. Elsharif S. A. Janzen D. Buettner A. Villmann C. (2017). Metabolic products of linalool and modulation of GABAA receptors. Front. Chem. 5 (JUN), 46. 10.3389/fchem.2017.00046 28680877 PMC5478857

[B52] Muradova M. Proskura A. Canon F. Aleksandrova I. Schwartz M. Heydel J. M. (2023). Unlocking flavor potential using microbial β-Glucosidases in food processing. Foods 12 (24), 4484. 10.3390/foods12244484 38137288 PMC10742834

[B53] Neto L. J. de L. Ramos A. G. B. de Freitas T. S. Barbosa C. R. D. S. de Sousa Júnior D. L. Siyadatpanah A. (2021). Evaluation of benzaldehyde as an antibiotic modulator and its toxic effect against drosophila melanogaster. Molecules 26 (18), 5570. 10.3390/molecules26185570 34577039 PMC8471095

[B54] Ning Y. Zhang L. Y. Mai J. Su J. E. Cai J. Y. Chen Y. (2023). Tobacco microbial screening and application in improving the quality of tobacco in different physical states. Bioresour. Bioprocess. 10 (1), 32. 10.1186/s40643-023-00651-6 38647749 PMC10992236

[B55] Pan S. P. Pirker T. Kunert O. Kretschmer N. Hummelbrunner S. Latkolik S. L. (2019). C13 megastigmane derivatives from epipremnum pinnatum: β-damascenone inhibits the expression of pro-inflammatory cytokines and leukocyte adhesion molecules as well as NF-κB signaling. Front. Pharmacol. 10, 1351. 10.3389/fphar.2019.01351 31849641 PMC6892967

[B56] Pan Y. Wang Y. Hao W. Duan C. Wang S. Wei J. (2022). Metatranscriptomics unravel composition, drivers, and functions of the active microorganisms in light-flavor liquor fermentation. Microbiol. Spectr. 10 (3), e02151-21. 10.1128/spectrum.02151-21 35638860 PMC9241730

[B57] Pandur E. Major B. Rák T. Sipos K. Csutak A. Horváth G. (2024). Linalool and geraniol defend neurons from oxidative stress, inflammation, and iron accumulation in *in vitro* parkinson’s models. Antioxidants 13 (8), 917. 10.3390/antiox13080917 39199163 PMC11351228

[B58] Parapouli M. Sfakianaki A. Monokrousos N. Perisynakis A. Hatziloukas E. (2019). Comparative transcriptional analysis of flavour-biosynthetic genes of a native *Saccharomyces cerevisiae* strain fermenting in its natural must environment, vs. a commercial strain and correlation of the genes’ activities with the produced flavour compounds. J. Biol. Research-Thessaloniki 26 (1), 5. 10.1186/s40709-019-0096-8 31406688 PMC6683356

[B59] Rodríguez-Bustamante E. Sánchez S. (2007). Microbial production of C13-norisoprenoids and other aroma compounds *via* carotenoid cleavage. Crit. Rev. Microbiol. 33 (3), 211–230. 10.1080/10408410701473306 17653988

[B60] Rodríguez-Medina N. Barrios-Camacho H. Duran-Bedolla J. Garza-Ramos U. (2019). Klebsiella variicola: an emerging pathogen in humans. Emerg. Microbes Infect. 8 (1), 973–988. 10.1080/22221751.2019.1634981 31259664 PMC6609320

[B61] Sabichi A. L. Xu H. Fischer S. Zou C. Yang X. Steele V. E. (2003). Retinoid receptor-dependent and independent biological activities of novel fenretinide analogues and metabolites. Clin. Cancer Res. 9 (12), 4606–4613. 14555536

[B62] Santos P. Busta L. Yim W. C. Cahoon E. B. Kosma D. K. (2022). Structural diversity, biosynthesis, and function of plant falcarin-type polyacetylenic lipids. J. Exp. Bot. 73 (9), 2889–2904. 10.1093/jxb/erac006 35560192

[B63] Sarry J. E. Günata Z. (2004). Plant and microbial glycoside hydrolases: volatile release from glycosidic aroma precursors. Food Chem. 87 (4), 509–521. 10.1016/j.foodchem.2004.01.003

[B64] Shakya M. Lo C. C. Chain P. S. G. (2019). Advances and challenges in metatranscriptomic analysis. Front. Genet. 10 (SEP), 904. 10.3389/fgene.2019.00904 31608125 PMC6774269

[B65] Shen X. Zhou M. Zhu X. Zhang J. Xu J. Jiang W. (2023). Chemical composition and antioxidant activity of petroleum ether fraction of Rosmarinus officinalis. Heliyon 9 (11), e21316. 10.1016/j.heliyon.2023.e21316 37942163 PMC10628691

[B66] Si H. Zhou K. Zhao T. Cui B. Liu F. Zhao M. (2023). The bacterial succession and its role in flavor compounds formation during the fermentation of cigar tobacco leaves. Bioresour. Bioprocess. 10 (1), 74. 10.1186/s40643-023-00694-9 38647588 PMC10992852

[B67] Silva R. Coelho E. Aguiar T. Q. Domingues L. (2021). Microbial biosynthesis of lactones: gaps and opportunities towards sustainable production. Appl. Sci. Switz. 11 (18), 8500. 10.3390/app11188500

[B68] Singh A. Vats S. Bhargava P. (2021). “Advances and challenges in metatranscriptomic analysis,” in Microbial metatranscriptomics belowground. 10.1007/978-981-15-9758-9_21

[B69] Siwach A. Verma P. K. (2020). Therapeutic potential of oxadiazole or furadiazole containing compounds. BMC Chem. 14 (1), 70. 10.1186/s13065-020-00721-2 33372629 PMC7722446

[B70] Stautz J. Hellmich Y. Fuss M. F. Silberberg J. M. Devlin J. R. Stockbridge R. B. (2021). Molecular mechanisms for bacterial potassium homeostasis. J. Mol. Biol. 433 (16), 166968. 10.1016/j.jmb.2021.166968 33798529 PMC9041122

[B71] Steinegger M. Söding J. (2017). MMseqs2 enables sensitive protein sequence searching for the analysis of massive data sets. Nat. Biotechnol. 35 (11), 1026–1028. 10.1038/nbt.3988 29035372

[B72] Suleria H. A. R. Gobe G. Masci P. Osborne S. A. (2016). Marine bioactive compounds and health promoting perspectives; innovation pathways for drug discovery. Trends Food Sci. Technol. 50, 44–55. 10.1016/j.tifs.2016.01.019

[B73] Suresh A. (2023). Oral microbial shift induced by probiotic Bacillus coagualans along with its clinical perspectives. J. Oral Biol. Craniofacial Res. 13 (3), 398–402. 10.1016/j.jobcr.2023.03.013 PMC10131115 37124834

[B74] Surowiak A. K. Balcerzak L. Lochyński S. Strub D. J. (2021). Biological activity of selected natural and synthetic terpenoid lactones. Int. J. Mol. Sci. 22 (9), 5036. 10.3390/ijms22095036 34068609 PMC8126056

[B75] Tao J. Chen S. Jiang Z. Wang C. Zhang E. Liang H. (2024). Fermentation process of tobacco leaves drives the specific changes of microbial community. BMC Microbiol. 24 (1), 534. 10.1186/s12866-024-03702-w 39716094 PMC11664857

[B76] Ullah I. Khan A. L. Ali L. Khan A. R. Waqas M. Hussain J. (2015). Benzaldehyde as an insecticidal, antimicrobial, and antioxidant compound produced by Photorhabdus temperata M1021. J. Microbiol. 53 (2), 127–133. 10.1007/s12275-015-4632-4 25626368

[B77] Wang S. N. Liu Z. Tang H. Z. Meng J. Xu P. (2007). Characterization of environmentally friendly nicotine degradation by Pseudomonas putida biotype A strain S16. Microbiology 153 (5), 1556–1565. 10.1099/mic.0.2006/005223-0 17464070

[B78] Wang Z. Gerstein M. Snyder M. (2009). RNA-Seq: a revolutionary tool for transcriptomics. Nat. Rev. Genet. 10 (1), 57–63. 10.1038/nrg2484 19015660 PMC2949280

[B79] Wang Y. Korneliussen T. S. Holman L. E. Manica A. Pedersen M. W. (2022). *ngsLCA*—A toolkit for fast and flexible lowest common ancestor inference and taxonomic profiling of metagenomic data. Methods Ecol. Evol. 13 (12), 2699–2708. 10.1111/2041-210X.14006

[B80] Wang D. Deng Y. Chen X. Wang K. Zhao L. Wang Z. (2023). Elucidating the effects of Lactobacillus plantarum fermentation on the aroma profiles of pasteurized litchi juice using multi-scale molecular sensory science. Curr. Res. Food Sci. 6, 100481. 10.1016/j.crfs.2023.100481 37033736 PMC10074505

[B81] Wang Y. Wang Y. Qiu S. Wang B. Zeng H. (2024a). Metagenomic and flavoromic profiling reveals the correlation between the microorganisms and volatile flavor compounds in Monascus-fermented cheese. Food Res. Int. 188, 114483. 10.1016/J.FOODRES.2024.114483 38823869

[B82] Wang Y. Zeng H. Qiu S. Han H. Wang B. (2024b). Identification of key aroma compounds and core functional microorganisms associated with aroma formation for Monascus-fermented cheese. Food Chem. 434, 137401. 10.1016/j.foodchem.2023.137401 37696158

[B83] Whited L. J. Hammond B. H. Chapman K. W. Boor K. J. (2002). Vitamin A degradation and light-oxidized flavor defects in milk. J. Dairy Sci. 85 (2), 351–354. 10.3168/jds.S0022-0302(02)74080-0 11913693

[B84] Yan T. Zhou P. Long F. Liu J. Wu F. Zhang M. (2022). Unraveling the difference in the Composition/content of the aroma compounds in different tobacco leaves: for better use. Genet. Res. 2022, 1–10. 10.1155/2022/3293899

[B85] Yuan Y. J. Lu Z. X. Huang L. J. Bie X. M. Lü F. X. Li Y. (2006). Optimization of a medium for enhancing nicotine biodegradation by Ochrobactrum intermedium DN2. J. Appl. Microbiol. 101 (3), 691–697. 10.1111/j.1365-2672.2006.02929.x 16907819

[B86] Yvon M. Rijnen L. (2001). Cheese flavour formation by amino acid catabolism. Int. Dairy J. 11 (4–7), 185–201. 10.1016/S0958-6946(01)00049-8

[B87] Zara G. Fan G. (2023). Editorial: microbial biotransformation of natural flavor compounds. Front. Microbiol. 14, 1243194. 10.3389/fmicb.2023.1243194 37497540 PMC10368183

[B88] Zhang Z. Mei X. He Z. Xie X. Yang Y. Mei C. (2022). Nicotine metabolism pathway in bacteria: mechanism, modification, and application. Appl. Microbiol. Biotechnol. 106 (3), 889–904. 10.1007/s00253-022-11763-y 35072735

[B89] Zhang C. L. Naicker O. Zhang B. Jin Z. W. Li S. J. Miao L. (2024). Transcriptome and hormonal analysis of Agaricus bisporus basidiome response to Hypomyces perniciosus infection. Plant Dis. 108 (2), 473–485. 10.1094/PDIS-05-23-0992-RE 37669175

[B90] Zheng R. Zhu Z. Wang Y. Hu S. Xi W. Xiao W. (2019). UGT85A84 catalyzes the glycosylation of aromatic monoterpenes in Osmanthus fragrans lour. Flowers. Front. Plant Sci. 10, 1376. 10.3389/fpls.2019.01376 31849999 PMC6902048

[B91] Zhu L. Zeng H. Liu D. Fu Y. Wu Q. Song B. (2020). Design, synthesis, and biological activity of novel 1,2,4-oxadiazole derivatives. BMC Chem. 14 (1), 68. 10.1186/s13065-020-00722-1 33292412 PMC7680602

[B92] Zou X. Bk A. Rauf A. Saeed M. Al-Awthan Y. S. Al-Duais M. A. (2021). Screening of polyphenols in tobacco (Nicotiana tabacum) and determination of their antioxidant activity in different tobacco varieties. ACS Omega 6 (39), 25361–25371. 10.1021/acsomega.1c03275 34632194 PMC8495694

[B93] Zou Y. Liu M. Lai Y. Liu X. Li X. Li Y. (2023). The glycoside hydrolase gene family profile and microbial function of Debaryomyces hansenii Y4 during South-road dark tea fermentation. Front. Microbiol. 14, 1229251. 10.3389/fmicb.2023.1229251 37502404 PMC10369063

[B94] Zou L. Zhang H. Liu Z. Sun J. Hu Y. Ding Y. (2024). Analyzing the effect of microbial consortia fermentation on the quality of HnB by untargeted metabolomics. J. Microbiol. Biotechnol. 34, 1890–1897. 10.4014/jmb.2402.02039 39187455 PMC11485560

